# Multifunctional Core-Shell Microgels as Pd-Nanoparticle Containing Nanoreactors With Enhanced Catalytic Turnover

**DOI:** 10.3389/fchem.2022.889521

**Published:** 2022-05-27

**Authors:** Viktor Sabadasch, Maxim Dirksen, Pascal Fandrich, Thomas Hellweg

**Affiliations:** Department of Physical and Biophysical Chemistry, Bielefeld University, Bielefeld, Germany

**Keywords:** microgels, responsive material, nanoparticles, catalysis, recycling, core-shell structure

## Abstract

In this work, we present core-shell microgels with tailor-made architecture and properties for the incorporation of palladium nanoparticles. The microgel core consists of poly-*N*-isopropylacrylamide (PNIPAM) copolymerized with methacrylic acid (MAc) as anchor point for the incorporation of palladium nanoparticles. The microgel shell is prepared by copolymerization of NIPAM and the UV-sensitive comonomer 2-hydroxy-4-(methacryloyloxy)-benzophenone (HMABP). The obtained core-shell architecture was analyzed by means of photon correlation spectroscopy, while the incorporated amount of HMABP was further confirmed *via* Fourier transform infrared spectroscopy. Subsequently, the microgel system was used for loading with palladium nanoparticles and their size and localization were investigated by transmission electron microscopy. The catalytic activity of the monodisperse palladium nanoparticles was tested by reduction of 4-nitrophenol to 4-aminophenol. The obtained reaction rate constants for the core-shell system showed enhanced activity compared to the Pd-loaded bare core system. Furthermore, it was possible to recycle the catalyst several times. Analysis *via* transmission electron microscopy revealed, that the incorporated palladium nanoparticles emerged undamaged after the reaction and subsequent purification process since no aggregation or loss in size was observed.

## Graphical Abstract

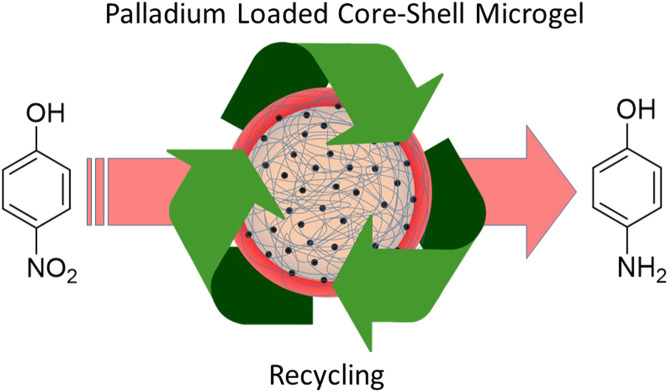

## 1 Introduction

The quest for more environmentally friendly and yet economical technologies is steadily driving new developments in catalysis chemistry. In particular, heterogeneous catalysis on nanoparticles (Wei et al., 2008; Wang et al., 2009; An and Somorjai, 2012; Dachwitz et al., 2020; Mohan et al., 2020; Seto et al., 2020) has received great interest since organic solvents and inert gas atmospheres are no longer necessary (Wong et al., 2009). The most studied nanoparticle systems are based on the transition metals Ag, Au, Pt, and Pd (Liu et al., 2015; Zhao et al., 2015; Bai et al., 2016; Brändel et al., 2019a). Although nanoparticles are highly catalytically active due to their large surface-to-volume ratio, new challenges arise due to their size. A major problem with nanoparticles is their difficult, residue-free separation from the product, which raises major concerns with regard to a green chemistry approach (Buzea et al., 2007; Elsaesser and Howard, 2012). Moreover, aggregation of nanoparticles can lead to another major challenge as it reduces the accessible surface area and catalytic activity (Ingham et al., 2011; Hansen et al., 2013). Both problems can be minimized by incorporating the nanoparticles into suitable carrier systems such as polyelectrolytes (Wunder et al., 2010; Battistoni et al., 2017; Du et al., 2019; Ding et al., 2021), inorganic nanocontainers (Zhang et al., 2009; Ding et al., 2011; Priebe and Fromm, 2014), or organic polyacrylamide-based microgels (Lu and Ballauff, 2011; Brändel et al., 2019b; Inui et al., 2020; Naseem et al., 2020; Oberdisse and Hellweg, 2020; Sabadasch et al., 2020; Iqbal et al., 2021). The latter systems offer a wide range of parameters to tune the catalytic activity of the embedded nanoparticles due to their versatile responsiveness triggered by temperature (Liu et al., 2009; Friesen et al., 2021), ionic strength (Karg et al., 2008) or pH (Snowden et al., 1996; Hoare and Pelton, 2004; Hoare and Pelton, 2006). In addition, the small size and high colloidal stability of these carrier systems enables a homogeneous distribution of the catalyst over the entire reaction volume. As a result, many systems exhibit superior catalytic activity. One of the best-known representatives of acrylamide-based microgels is PNIPAM, which shows a so-called volume phase transition temperature (VPTT) of 33 °C, accompanied by a drastic decrease in particle size (Wu et al., 1996; Wrede et al., 2018). The properties of these microgels can be tuned very precisely, e.g., by using hydrophobic or hydrophilic comonomers, (Anakhov et al., 2020), adding surfactants (Wedel et al., 2017) or realizing special particle architectures such as core-shell particles (Berndt. and Richtering., 2003; Hellweg et al., 2004; Berndt et al., 2006; Häntzschel et al., 2007; Cors et al., 2017; Ghavami and Winkler, 2017). Core-shell particles stand out due to different chemical structure of the outer and inner regions. In a previous work, we were able to load a core-shell system consisting of a polyacrylamide core copolymerized with MAc and an additional acid free shell (Sabadasch et al., 2020). While the microgel core offered the anchor points for the incorporation of nanoparticles, the acid free shell led to a temperature induced switchability of the catalytic activity of the embedded catalyst. In the present work, we targeted a polyacrylamide-based core-shell system as a carrier system for palladium nanoparticles. Therefore, we applied a PNIPAM shell with an additional UV-sensitive comonomer HMABP onto previously prepared PNIPAM core particles with methacrylic acid as comonomer (Sabadasch et al., 2022). HMABP was used as UV-sensitive monomer as it enables a post-synthetic cross-linking of the microgel particles. In a recent study, we were able to use this comonomer to prepare free-standing microgel films. The prepared films can be transferred onto various substrates or can be used as free-standing diffusion barriers (Dirksen et al., 2022). The incorporation of HMABP was characterized by means of PCS and Fourier transform infrared spectroscopy (FTIR). After loading the core-shell system with palladium nanoparticles, the localization and size of the nanoparticles were characterized *via* transmission electron microscopy (TEM). Catalytic activity of the loaded microgel system was investigated by a reduction of 4-nitrophenol to 4-aminophenol as a model reaction (Lu and Ballauff, 2011). By straightforward purification *via* centrifugation, it was possible to recycle the catalyst several times. The combination of TEM imaging and kinetic data obtained from UV-VIS measurements reveal detailed insight into the properties of the recycled nanoparticles.

## 2 Materials and Methods

### 2.1 Materials

*N*-Isopropylacrylamide (NIPAM, TCI Germany GmbH, Eschborn, Germany; 97%) was recrystallized from *n*-hexane. *N,N′*-methylenebisacrylamide (BIS, Sigma-Aldrich, Munich, Germany; 99%), sodium dodecyl sulfate (SDS, Sigma-Aldrich, Munich, Germany; > 99.5%), ammonium peroxodisulfate (APS, Sigma-Aldrich, Munich, Germany; ≥ 98%), 2-hydroxy-4-(methacryloyloxy)-benzophenone (HMABP, Alfa Aesar, Karlsruhe, Germany; 99%), PdCl_2_ (Sigma-Aldrich, Munich, Germany; > 99.9%), ammonia (Carl Roth, Karlsruhe, Germany; 25%), HCl (0.1 M, Fischer Scientific GmbH, Schwerte, Germany, volumetric solution), NaOH (0.1 M, Fischer Scientific GmbH, Schwerte, Germany, volumetric solution), NH_4_Cl (Carl Roth, Karlsruhe, Germany; ≥ 99.5%), acetic acid (VWR International, Eschborn, Germany; 100%), sodium acetate trihydrate (Sigma-Aldrich, Munich, Germany; ≥ 97%) and sodium borohydride (Sigma-Aldrich, Munich, Germany; 98.0%) were used without further purification. Water was purified using an Arium pro VF system (Sartorius AG, Göttingen, Germany).

### 2.2 Microgel Synthesis

The core-shell microgels were synthesized in a two-step precipitation polymerization following the typical synthesis route published by Pelton and Chibante (Pelton and Chibante, 1986). The PNIPAM-*co*-MAc core particles were prepared as described previously (Sabadasch et al., 2022). For the shell-synthesis a modified seeded precipitation polymerization was performed (Zeiser et al., 2012). Therefore, NIPAM (2.06 mmol), BIS (0.13 mmol, 5 mol%) and SDS (0.07 mmol) were added to a dispersion of the core-microgel (49 ml, 0.15 wt%) in purified water and equillibrated like in the core synthesis. Simultaneously HMABP (0.103  mmol, 5 mol%) was dissolved in 5 ml of the reaction solution, treated with an ultrasonic bath and added to the reaction mixture. The polymerization was initiated by adding APS (0.14  mmol, 6.6 mol%) dissolved in 1 ml of purified water. In analogy to the core synthesis, the core-shell synthesis mixture was refluxed for 4 h and purified by centrifugation.

### 2.3 Titration

A defined mass of the respective microgel suspension was acidified with hydrochloric acid (0.1 M) to obtain a defined fully protonated state of the respective microgel. The suspension was then titrated against sodium hydroxide (0.1 M) in increments of 6 µl every 5 s at a temperature of (23 ± 1) °C with a Metrohm 905 automatic titrator (Metrohm, Herisau, Schweiz). The volume difference between the equilibrium points of the excess hydrochloric acid and the copolymerized acidic moieties was used to calculate the acid content per dry mass of polymer *n*_MAc_. The half equivalence point between the neutralisation of excess hydrochloric acid and copolymer correspond to the apparent p*K*_a_ value of the copolymer. Both microgel systems were titrated three times. The derived values for the apparent p*K*_a_ and the acid content *n*_MAc_ were mediated and are listed in Supplementary Table S1.

### 2.4 Attenuated Total Reflection Fourier Transform Infrared Spectroscopy

ATR-FTIR measurements were performed at an IFS 66/S FTIR spectrometer (Bruker, Ettlingen, Germany) with a mercury cadmium telluride detector and a diamond/ZnSe internal reflection element with nine active reflections (DuraSamplIR II, Smiths, CT, United States). The spectra were obtained from 512 scans with a resolution of 2 cm^−1^. The microgel suspension (20 μl, > 0.4 wt%) was deposited onto the crystal and dried with air for at least 30 min. The absorbance of the corrected spectra was multiplied with the corresponding wavenumber to compensate the wavenumber dependent penetration depth and was normalized to the amide I vibration at 1,642 cm^−1^.

### 2.5 Incorporation of Palladium Nanoparticles Into Microgels

A core-shell microgel suspension with a mass fraction of 0.04% with regard to the final reaction suspension mass of 50 g was given into a flask, purged with nitrogen and stirred with a magnetic stirrer. Subsequently a PdCl_2_ solution (532 μl, 20 mM PdCl_2_ with 800 mM ammonia) was added and the suspension was cooled with an ice bath resulting in a pH value of 11.3. After an equilibration time of 1 hour a NaBH_4_ solution (5 ml, 3.2 M) was added. The ice bath was removed and upon equilibrating to room temperature the suspension was filtered over glass wool. The product was filled in a dialysis tube (Spectra/Por Biotech CE tubing 1,000 kDa, Repligen, Waltham, MA, United States), placed in a beaker with a volume of 5 L and dialyzed over 36 h against water. The water was exchanged four times within this time range. The mass of incorporated palladium per suspension mass in the core-shell hybrid system was determined by atomic absorption spectroscopy (AAS) by Mikroanalytisches Laboratorium Kolbe (Oberhausen, Germany). Since the suspension was highly diluted, a density of 1 g ml^−1^ was assumed. The mass of palladium per reaction volume for the prepared core-shell/palladium hybrid system was *m*_r_ = (29 ± 1) μg ml^−1^.

### 2.6 Photon Correlation Spectroscopy

For the sample preparation all microgel suspensions were highly diluted with buffer solutions consisting of acetic acid/sodium acetate (5 mM, pH 4) and ammonia/ammonium chloride (5 mM, pH 10), respectively. To determine the particle size of the microgels, angle-dependent PCS measurements were performed using a 3D-LS Spectrometer Pro (LS Instruments AG, Fribourg, Switzerland) equipped with a HeNe laser (1145P, JDS Uniphase Corp. Milpitas, CA, United States). The temperature was adjusted to 20 °C using a thermostated decaline index matching bath with an equilibration time of 20 min at the desired temperature. For each sample three measurements in an angular range from 40 ° to 120° in steps of 5° were performed.

For the temperature-dependent determination of the hydrodynamic radius a setup consisting of a HeNe laser (HNL210L, Thorlabs Inc. Newton, NJ, nited States) with an ALV-6010 multiple-*τ* correlator (ALV GmbH, Langen, Germany) was used. The measurements were performed at a constant angle of 45° in a temperature range of 10–60 °C. The temperature was adjusted using a thermostated decaline index matching bath. At each temperature the sample was allowed to equilibrate for 25 min. The resulting auto-correlation functions were analyzed using the method of cumulants to obtain the mean relaxation rates $\bar{\Gamma }$ (Provencher, 1982). *Via* $\bar{\Gamma }=D^{\text{T}}\cdot q^{2}$ the translational diffusion coefficient *D* ^T^ can be calculated using the magnitude of the scattering vector $q=|\vec{q}|=\frac{4\pi n}{\lambda }\cdot \sin\left(\frac{\theta }{2}\right)$ . Here, *λ* is the wavelength of the scattered light, *n* the refractive index of the solvent and *θ* the scattering angle. The hydrodynamic radius *R*_h_ of the microgel particles can be calculated using the Stokes-Einstein equation,

$$D^{\text{T}}=\frac{k_{\text{B}}T}{6\pi \eta R_{\text{h}}}$$ (1)

with the Boltzmann constant *k*_B_, the temperature *T* and *η* the viscosity of the sample.

### 2.7 Transmission Electron Microscopy

For transmission electron microscopy carbon-coated copper grids (CF200-Cu, Electron Microscopy Sciences, Hatfield, PA, United States) were used. Prior the preparation, the copper grids were treated with argon plasma using a plasma cleaner (Zepto, Diener Electronics, Ebhausen, Germany). The samples were prepared by applying 3 *μ*L of the microgel suspension onto the copper grid. After a sedimentation time of 1 minute, most of the residual suspension was removed with a filter paper. The images were taken on a JEOL JEM-2200FS electron microscope (JEOL, Freising, Germany) at an acceleration voltage of 200 kV. The microscope was equipped with a cold field emission electron gun. The digital recording of the images was carried out by a bottom-mounted Gatan OneView camera (Gatan, Pleasanton, CA, USA) and the images were further processed with the digital imaging processing system Digital Micrograph GMS3 (Gatan, Pleasanton, CA, United States). The nanoparticle size analysis was performed with the image editing software ImageJ (Schneider et al., 2012). Combining the mass of palladium per reaction volume *m*_r_ with the mean diameter *d*_NP_ of the particles the total surface area per reaction volume *S*_r_ was calculated:

$$S_{\text{r}}=N_{\text{NP}}\cdot A_{\text{NP}}=N_{\text{NP}}\cdot {d_{\text{NP}}}^{2}\cdot \pi .$$ (2)

*N*_NP_ is the number of palladium nanoparticles per reaction volume, the surface area a single particle provides is *A*_NP_. The number of palladium nanoparticles per reaction volume *A*_NP_ is defined by:

$$N_{\text{NP}}=m_{\text{r}}\cdot {V_{\text{NP}}}^{-1}\cdot \rho^{-1}=m_{\text{r}}\cdot {\left(\frac{1}{6}\cdot \pi \cdot {d_{\text{NP}}}^{3}\right)}^{-1}\cdot \rho^{-1}$$ (3)

where *ρ* is the bulk density of palladium (12.0 g cm^−3^) (Lide, 2004) and *V*_NP_ is the volume of a single nanoparticle.

### 2.8 UV-VIS Spectroscopy

The UV-VIS spectroscopic measurements were performed on an Agilent 8453 spectrometer (Agilent Technologies, Rattingen, Germany). The spectrometer was equipped with an eight-position holder and a diode array detector system. The cuvette holder was thermostated with a Julabo F25 thermostat (Julabo GmbH, Seelbach, Germany). The catalytic activity of the free hybrid core-shell microgel system was tested *via* the reduction of 4-nitrophenol to 4-aminophenol with an excess of sodium borohydride in presence of the catalyst at 20°C. The catalyst (100 µl) was given into the cuvette (Hellma Analytics, Mülheim, Germany, 10 mm path length, QS standard) and was diluted with water (100 µl). The sodium borohydride (50 μl, 300 mM) was dissolved in water and was added to the catalyst suspension. After an equilibration time of 3 minutes the 4-nitrophenol solution (750 μl, $0.0\bar{6}$ M) was added, resulting in a final educt concentration of 0.05 mM and reducing agent concentration of 15 mM. For recycling, the suspension was transferred into a centrifuge tube and diluted to a volume of 2 ml. The sample was centrifuged for 20 min at 21,400 g at 20 °C. Afterwards 1800 µl of supernatant were removed, the suspension was redispersed with water and centrifuged again. Finally 1800 µl of the supernatant were removed and the remaining pellet was redispersed with a sodium borohydride solution (50 μl, 300 mM). Subsequent cycles were started upon addition of a nitrophenol solution (750 μl, $0.0\bar{6}$ M). The experiments were performed twice, and derived data were mediated (Supplementary Table S2, ESI).

## 3 Results

### 3.1 Microgel Characterization

The present core and core-shell particles were synthesized *via* precipitation polymerization (Pelton and Chibante, 1986). In this process, previously prepared PNIPAM core particles copolymerized with methacrylic acid (Sabadasch et al., 2022) were used as seed particles for the synthesis of the core-shell microgels containing the UV-sensitive comonomer HMABP which allows further cross-linking of the particles to form e.g., membranes (Dirksen et al., 2021). The apparent p*K*_a_ values for the copolymerized acidic groups in the core and core-shell particles were determined by titration to be approx. 6.6 and 6.5, respectively (Supplementary Table S1, ESI). The increased p*K*_a_ value for the copolymerized methacrylic acid [monomer p*K*_a_ = 4.66 (Seidel et al., 2007)] in the polymer network can be attributed to the polyelectrolyte effect of the neighboring acidic groups, which influence the acidity. The data obtained here also nicely agree with the observations for PNIPAM-*co*-methacrylic acid microgels by other groups. (Hoare and Pelton, 2004; Kleinen and Richtering, 2011). Accordingly, the pH values of 4 and 10 correspond to the fully protonated and deprotonated state of copolymerized methacrylic acid.

**FIGURE 1:**
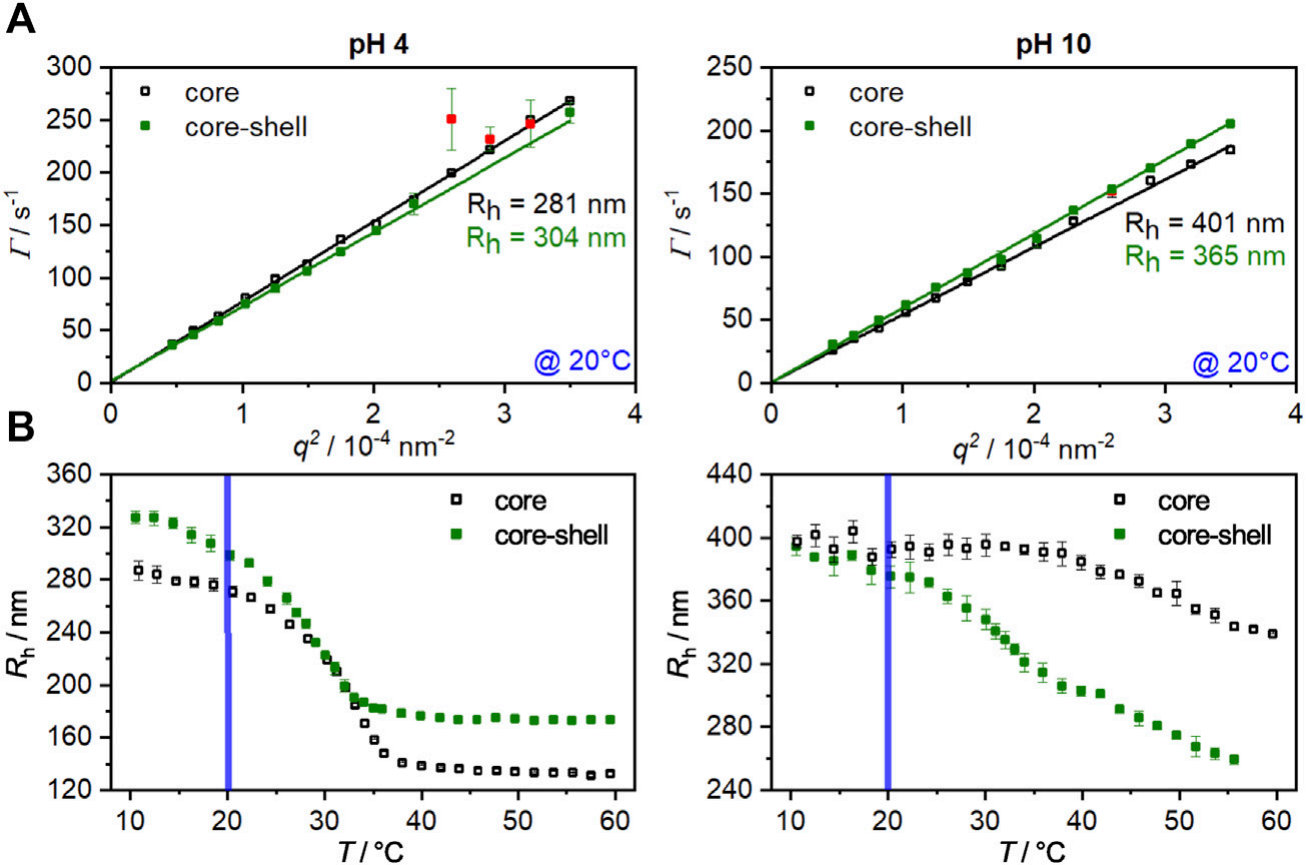
**(A)**: Angle-dependent PCS measurements for the core (hollow squares) and core-shell particles (filled squares) at pH 4 and pH 10 in the swollen state (20°C). The slopes obtained from the linear fits were used to calculate the hydrodynamic radius by the Stokes-Einstein equation. Red data points were masked and not used for the fitting procedure. **(B)**: Hydrodynamic radii of the core and core-shell microgels as function of temperature. The measurements were performed at a fixed angle at pH 4 and pH 10. The blue line represents the temperature at which the angle-dependent measurement were performed. The error bars correspond to the standard deviation.

To investigate the hydrodynamic properties of the core and core-shell particles angle-dependent photon correlation spectroscopy (PCS) measurements in the swollen state (20°C) at fully protonated (pH 4) and deprotonated (pH 10) conditions were performed. The results are shown in Figure 1A. All measurements led to a linear relation between the relaxation rate $\bar{\Gamma }$ and *q* ^2^ exhibiting an intercept through the origin which indicates the absence of rotational or deformation contributions. At a pH of 4, the MAc is present in the completely protonated state. Looking at the calculated radii for the core particles (281 nm) and core-shell particles (304 nm), rather weak increase in size is observable. When the pH value is increased (pH 10), the MAc is deprotonated, as a result of which the core particles are significantly larger compared to the protonated state due to a larger solvent sphere (Martinez-Moro et al., 2020). Despite being bigger at pH 4, the core-shell particles are smaller than the core at pH10. The pH-induced swelling is thus minimized due to the additional shell (Brändel et al., 2019a; Sabadasch et al., 2020).

In order to investigate the influence of the copolymerization of methacrylic acid and the UV-sensitive comonomer on the swelling behavior of the microgels, temperature-dependent PCS measurements for the core and the core-shell system at pH values of 4 and 10 were performed (Figure 1B).

At a pH of 4, the core collapses from a radius of about 280 nm in the swollen state (10°C) to a radius of about 135 nm at 60°C. Since the methacrylic acid is completely protonated at this pH value, a VPTT of 33°C is obtained. This corresponds exactly to the VPTT of PNIPAM homopolymer microgels (Kojima, 2018; Friesen et al., 2021). When the pH is increased to 10, the methacrylic acid is deprotonated, resulting in an increase in hydrophilicity of the system due to the anionic carboxylate groups, which leads to a strong broadening of the phase transition and a shift in the VPTT to higher temperatures (Martinez-Moro et al., 2020). By applying a shell to the core, a significant increase in the hydrodynamic radius of the particles at pH 4 in the swollen and collapsed states can be registered. Furthermore, a shift of the VPTT to 28°C occurs, which is related to the successful incorporation of the hydrophobic comonomer HMABP. It is not uncommon for copolymerization of a hydrophobic comonomer to lead to this effect (Kaneko et al., 1995; Zhang and Zhuo, 2002; Hertle et al., 2010; Okudan and Altay, 2019; Bookhold et al., 2021). Specifically for the comonomer used here, we were able to show a significant shift in VPTT to lower temperatures in a recent article (Dirksen et al., 2021).

Increasing the pH to 10 leads to almost equal hydrodynamic radii of the core and the core-shell particles in the swollen state. At this point it becomes clear that the pH-induced swelling of the core is inhibited by the non-pH responsive shell. Only at a temperature of 20°C, as already seen in the angle-dependent measurements, a minor difference in size is observed, indicating the onset of shell collapse. Furthermore, the swelling curve shows two distinct phase transitions at this pH value. The phase transition at approx. 33°C occurs due to the collapse of the shell. The core, which shows a significantly higher VPTT under basic conditions, collapses only at higher temperatures, and a fully collapsed state is not yet reached at 60°C. While the pure core system collapses up to a hydrodynamic radius of about 340 nm, the core-shell system collapses to a significantly smaller radius of 260 nm. At first glance, it seems counter-intuitive that a core system is bigger than the corresponding core-shell system, but several factors must be considered here. Upon growing a polyacrylamide-based shell onto a polyacrylamide core particle the core and shell region are not sharply separated from each other as shown by a recent work of Cors et al. Depending on polyacrylamide combination, a significant volume increment of shell-material can interpenetrate the core and shifts the VPTT of the respective region (Cors et al., 2019). In this case the hydrophobic comonomer shifts the VPTT towards far lower temperatures, so that the volume phase transition of the core-shell particle is more prominent than in the core system. Additional measurements via ATR-FTIR confirm a successful incorporation of HMABP into the core-shell system (Supplementary Figure S1, ESI) (Bookhold et al., 2021). From the obtained results, it can be summarized that the microgels exhibit pH sensitivity due to the copolymerization of methacrylic acid and that the incorporation of the comonomer HMABP into the microgel shell was successful.

### 3.2 Nanoparticle Characterization

**FIGURE 2:**
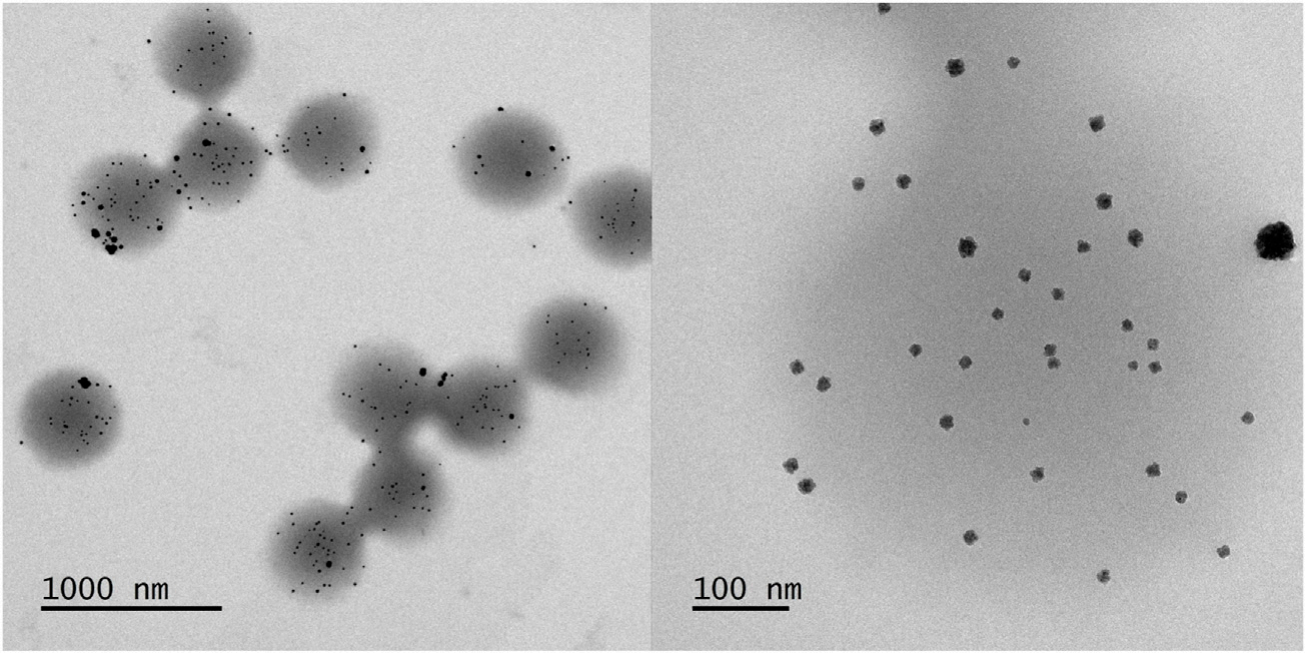
TEM micrographs of the core-shell microgels containing Pd nanoparticles. Two different magnifications with the respective scale bars are shown.

**FIGURE 3:**
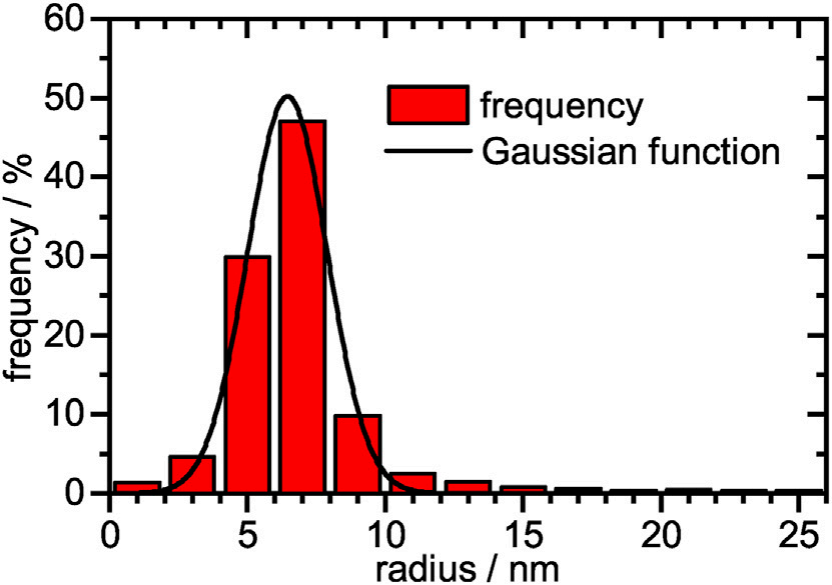
Histogram of the relative frequencies plotted against the nanoparticle radius. The size distribution was fitted with a single Gaussian function. A total of 2,300 palladium nanoparticles were analyzed. A mean particle radius of (6.4 ± 1.4) nm was obtained.

Subsequently the core-shell PNIPAM microgels were loaded with palladium nanoparticles. For this purpose, the microgel suspension was mixed with a Pd(NH_3_)_4_^2+^ solution. Due to the containing ammonia, the resulting suspension had a pH of 11.3, which induces a full deprotonation of the copolymerized methacrylic acid, so that a large number of negatively charged anchor points for the positively charged palladium complex were present. Reduction of the Pd^2+^ with sodium borohydride yields a microgel/palladium hybrid suspension with a palladium mass per volume of *m*_r_ =(29 ± 1) μg/ml. TEM micrographs can be seen in Figure 2. The monodisperse microgels with a slightly grey contrast show a variety of highly contrasted palladium nanoparticles incorporated inside the polymer network. A magnified view of a single microgel (Figure 2, right) reveals that the nanoparticles show a slight raspberry-like morphology. The mean nanoparticle radius is determined in a statistical analysis to be (6.4 ± 1.4) nm (Figure 3). The vast majority of nanoparticles are distributed randomly throughout the microgel and have a narrow size distribution. Knowing the concentration of palladium per volume and size of the nanoparticles, the surface area per reaction volume *S*_r_ = 0.113 m^2^l^−1^ can be calculated (see experimental section 2.7).

### 3.3 Catalytic Activity

**FIGURE 4:**
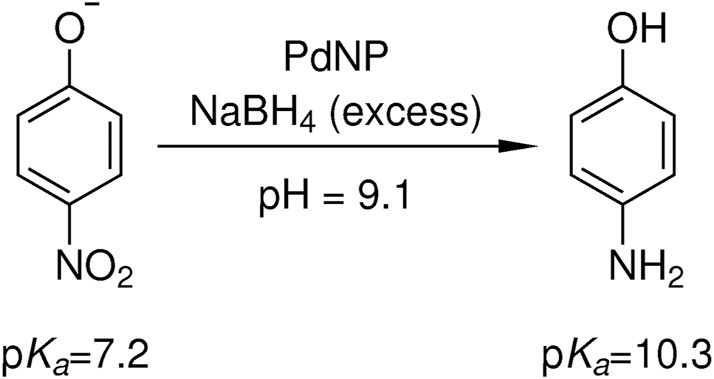
Catalytic reduction of 4-nitrophenolate to 4-aminophenol with an excess of sodium borohydride in the presence of palladium nanoparticle containing core-shell microgels. Due to the hydrolysis of sodium borohydride, an initial pH value of 9.1 is present and the product 4-aminophenol with a p*K*_a_ of 10.3 remains in its protonated state (Haynes, 2017).

To investigate the catalytic activity of the embedded palladium nanoparticles, the reduction of 4-nitrophenol to 4-aminophenol with sodium borohydride as reducing agent was chosen as an established model reaction (Figure 4) (Wunder et al., 2010). Since the catalytic reduction takes place at elevated pH values, due to the reducing agent, the deprotonated 4-nitrophenolate gives a characteristic absorbance maximum at 400 nm, while the product 4-aminophenol gives an absorbance maximum at about 300 nm. In the last 2 decades, this reaction has received great attention in the nanoparticle community since the decay of 4-nitrophenolate to 4-aminophenol can easily be studied by UV-VIS spectroscopy (Lu et al., 2007; Wunder et al., 2011; Hervés et al., 2012). If the reducing agent sodium borohydride is used in a high excess to the reactant, the reaction follows a pseudo-first order kinetic.

$$A=A_{0}\cdot \exp\left(-k_{\text{app}}\cdot t\right)+A_{\text{i}}$$ (4)

Where *A* and *A*_0_ are the absorbances at the times *t* and *t*_0_. The apparent rate constant is defined by *k*_app_ and the residual absorbance intensity, contributed from the microgel, is described with *A*_i_.

**FIGURE 5:**
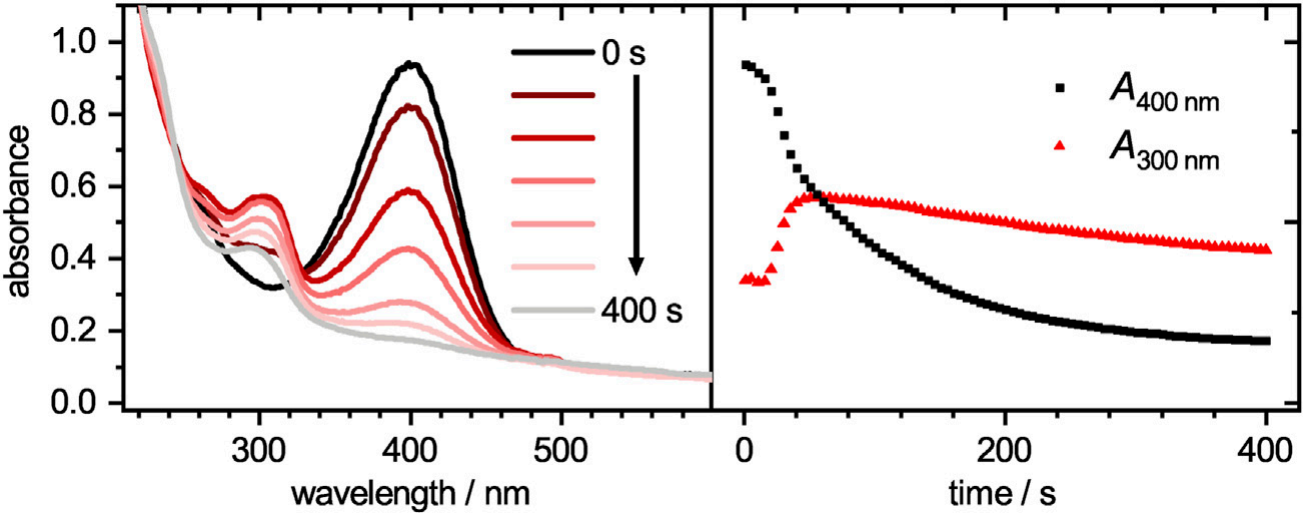
Absorbances plotted against the wavelength (left) for the catalytic degradation of 4-nitrophenolate to 4-aminophenol with palladium loaded microgels as catalyst at different times. The time-dependent absorbance intensities at wavelengths of 300 and 400 nm are plotted on the right. While 4-nitrophenolate, which absorbs mainly at 400 nm, decays, the product 4-aminophenol at 300 nm arises. Since the microgel has a wavelength-dependent scattering, the baseline intensity increases with decreasing wavelength.

The catalytic activity of the core-shell system was analyzed by tracing the decay of 4-nitrophenolate upon adding it to a mixture of the core-shell microgel system and sodium borohydride. Selected spectra for the catalytic reduction of 4-nitrophenolate at different times *t* are plotted in Figure 5 (left). A progressive decay of 4-nitrophenolate (400 nm) can be clearly seen in the time-dependent plot in Figure 5 (right) and be parsed in three regions. Initially, a short plateau region is observed. A common feature of heterogeneous catalyst, which is attributed to a restructuring of the palladium surface to its catalytic active form (Kaiser et al., 2012). It is followed by a fast decay which can be discussed with the initially fast reaction of 4-nitrophenolate to intermediate products like 4-nitrosophenol, 4-hydroxylaminophenol and 4,4′-dihydroxyazobenzene (Gu et al., 2014; Roa et al., 2018; Besold et al., 2021). After the intermediates reach a stationary state of adsorption, desorption and conversion, the decay transits into a third region. This region can be fitted with a pseudo-first order kinetic (Gu et al., 2014).

**FIGURE 6:**
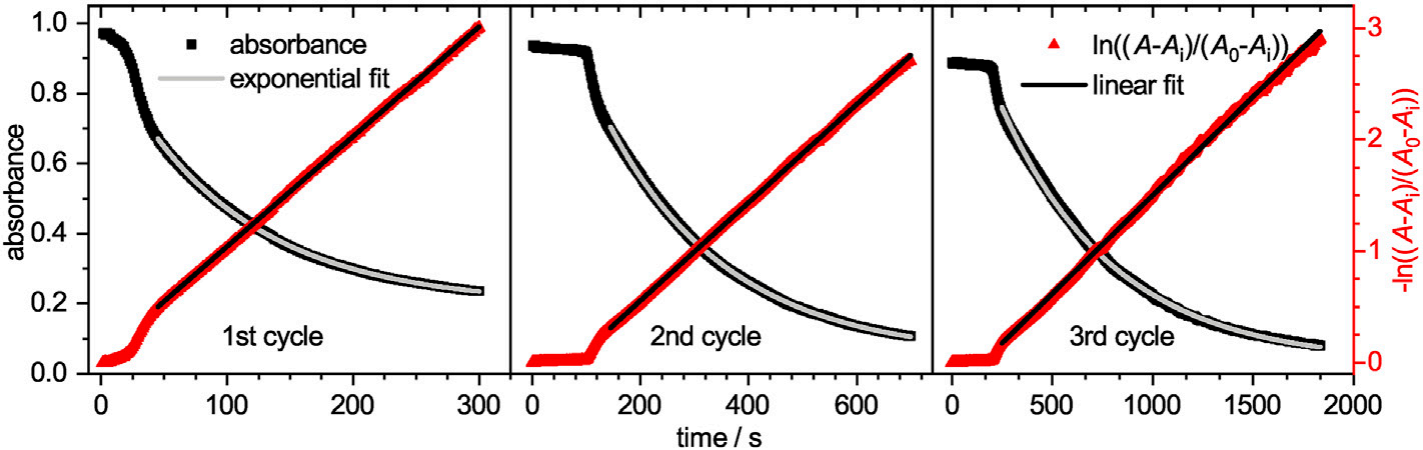
On the left y-axis the absorbance at a wavelength of 400 nm is plotted against the time for the three catalytic cycles. Each cycle has an own horizontal axis scaling, while all three share the same ordinate axis. The shown decay represents a conversion up to 95%. A mono-exponential pseudo-first order fit was applied (solid red line). The resulting baseline absorbance *A*_i_ and the initial absorbance *A*_*0*_ were applied for the calculation of ln ((*A*− *A*_i_)/(*A*_0_− *A*_i_)). On the right ln ((*A*− *A*_i_)/(*A*_0_− *A*_i_)) is plotted against the time and fitted with a straight line (solid black line) in the respective area.

The time-dependent absorbance intensity at 300 nm is also plotted in Figure 5. Similar to the observation for the absorbance band at 400 nm, likewise three distinct regions can be identified. The initial plateau region is followed by a steep increase, which transits into a slow intensity decrease. All three regions appear at similar time scales as the previously observed regions at 400 nm. The initial plateau region, where no reaction occurs is reconfirmed as no product is formed during this period. The second region, where intermediates are mainly formed lead to a steep increase in intensity. Although 4-hydroxylaminophenol and 4,4′-dihydroxyazobenzene are both converted to 4-aminophenol as last catalytic step, both contribute to the detected absorbance intensity. In the case of the 4,4′-dihydroxyazobenzene, it was shown to have an absorbance maximum at 300 nm, as does the 4-aminophenol product, but its molar extinction coefficient is significantly higher (Besold et al., 2021). Since those intermediates are converted to 4-aminophenol with a lower molar extinction coefficient, the absorbance intensity decreases in the third region. After the reaction was completed, the catalyst was purified as stated in the experimental section and recycled for two more cycles. The absorbance decays are plotted in Figure 6. In the region, where the absorbance follows a mono-exponential decay, it was fitted with a pseudo-first order kinetic model (Eq.  4). The baseline absorbance *A*_i_ derived from the fit was used to plot -ln ((*A*− *A*_i_)/(*A*_0_ − *A*_i_)) against the time. Fitting the linear region gives the apparent reaction rate constant *k*_app_ (Supplementary Table S2, ESI).

**FIGURE 7:**
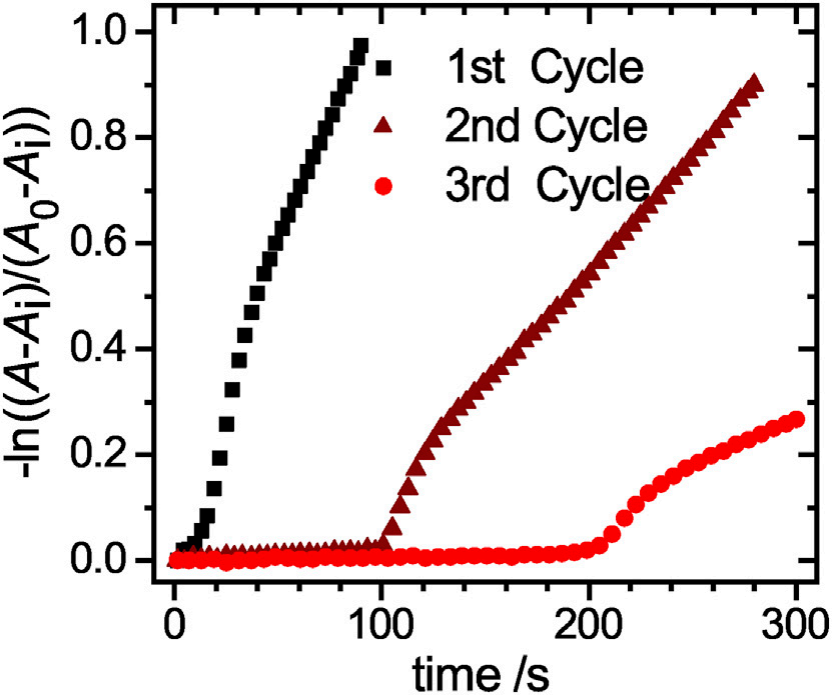
Magnified view of the linearized absorbance decay at a wavelength of 400 nm for the three cycles of the free hybrid system reactions. The three distinct regions of the catalytic reaction can be clearly identified.

The reaction rate *k*_app_ decreases significantly from cycle to cycle. While in the 2nd cycle only half of the reaction rate is reached, the value drops to less than one fifth of the initial value in the 3rd cycle. This significant loss is mainly caused by the loss of microgel during the purification process, indicated by the dropping residual absorbance intensity *A*_i_ (Supplementary Table S2). To investigate the early stages of the catalysis in detail, a magnified view of the linearized absorbance intensities is plotted in Figure 7. The initial induction time increases significantly from cycle to cycle, up to a value of approx. 120 s for the 2nd cycle and 210 s for the 3rd cycle. The increased time the catalyst requires to regain its catalytic activity, can be explained by processes like poisoning of the catalyst surface or hindered diffusion due to clogging of the network with product. To exclude that the reduced amount of catalyst in the 2nd and 3rd cycle causes this effect, we performed reactions with reduced amounts of catalyst. In Supplementary Figue  S2 the absorbance at 400 nm is plotted against the time for a variation of catalyst concentrations. The period in which no product is formed increases only insignificantly, so that the prolonged induction time observed during recycling is not caused by the loss of the catalyst. Additional TEM analysis of the nanoparticles after the first reaction and purification (Supplementary Figures S3, S4, ESI) further validate, that the nanoparticles with a mean size of (6.5 ± 1.5) nm retain their size and no aggregation or loss in size occurred.

The reaction rate constants *k*_app_ derived from the first cycle was further normalized towards the used palladium surface per reaction volume *S*_r_ to obtain *k*_norm._ (Table 1). This normalized reaction rate constant can be compared with incorporated palladium nanoparticles in other carrier systems. We previously incorporated palladium nanoparticles into a PNIPAM core system, which was used here as seed particles for the preparation of the core-shell system. The catalytic activity was equally tested as for the core-shell/Pd hybrid system and a normalized reaction rate was calculated (Table 1). Compared to the loaded core system, the core-shell system shows an increase in catalytic activity of about 29%. A similar observation was previously made upon applying an acid free shell onto poly-*N*-*n*-propylacrylamide microgel cores (Sabadasch et al., 2020). Likewise as stated in the previous study the catalytic activity of palladium nanoparticle was improved upon altering the polymer composition of the microgel by applying an additional microgel shell consisting of PNIPAM-*co*-HMABP. In this way the catalytic activity of the embedded nanoparticles was greatly improved. It is likely that the added shell reduces the amount of MAc copolymer per polymer mass, thus reducing the electrostatic repulsion between negatively charged 4-nitrophenolate and MAc carboxylates in the core-shell system. Kalekar et al. previously demonstrated the preparation of freely dispersed palladium nanoballs, which were more or less spherical porous palladium structures with a size of up to 90 nm (Kalekar et al., 2016). They tested their catalytic activity with the reduction of 4-nitrophenolate to 4-aminophenol and the normalized reaction rate constant of that system is listed in Table 1. The system shows an almost 4-fold faster reaction rate as compared to the core-shell system. It is commonly observed that the catalytic activity of nanoparticles confined in less cross-linked structures like dendrimers or polyelectrolytes has up to an order of magnitude higher activity compared to nanoparticles entrapped in acrylamide-based microgels (Mei et al., 2007). This can mainly be attributed to the reduced diffusion rate of reactants within the highly cross-linked gel material (Angioletti-Uberti et al., 2015).

**TABLE 1:** Apparent reaction rate constants *k*_app_ were normalized towards the palladium mass per reaction volume *S*_r_ to obtain *k*_norm_. The catalytic activity of a previously prepared PNIPAM-*co*-MAc core particle (Sabadasch et al., 2022) was analyzed analogously to the core-shell system. In addition, data for the reduction of 4-nitrophenolate to 4-aminophenol with palladium nanoballs (PdNBs) were derived from literature (Kalekar et al., 2016). All data refer to experiments at  20°C.

sample	*k*_app_/10^−3^ *s* ^−1^	*S*_r_/m^2^L^−1^	*k*_norm_/10^–2^ s^−1^m^−2^L
Core	14.1 ± 0.6	0.223	6.3 ± 0.2
core-shell	9.2 ± 0.7	0.113	8.1 ± 0.6
PdNBs	—	—	32 ± 1

## 4 Conclusion

In this study, we successfully prepared multifunctional core-shell microgels *via* seeded precipitation polymerization. The incorporation of the UV-sensitive comonomer HMABP in the microgel shell was confirmed by means of PCS and FTIR, providing further information about the microgel architecture. Based on the strong shift of the core-shell VPTT to lower temperatures and the two-step phase transition, not only a strongly interpenetrated region of core and shell material but also the existence of a region, consisting of shell material only, can be confirmed. By subsequently loading the core-shell system with catalytically active palladium nanoparticles with a size of (6.4 ± 1.4) nm, the core-shell system studied here shows enhanced catalytic activity compared to the loaded core system (Sabadasch et al., 2022). The kinetic study of several cycles prove that, in general, a recycling is possible. Due to the successful incorporation of the UV-sensitive HMABP comonomer, the core-shell/Pd hybrid system offers further potential concerning the preparation of catalytic active membranes, which will be presented in the future.

## Data Availability

The raw data supporting the conclusion of this article will be made available by the authors, without undue reservation.
