# Supplementary material for: Multifunctional Core-Shell Microgels as Pd-Nanoparticle Containing Nanoreactors With Enhanced Catalytic Turnover
Source: Front Chem. 2022 May 27;10:889521. doi: 10.3389/fchem.2022.889521 (PMC9185801; doi:10.3389/fchem.2022.889521)
Supplement: Supplementary file 1 [file DataSheet1.pdf]

Supplementary Material

Table S1. Methacrylic acid content n_{MAc} per dry mass of polymer and apparent pK_a value for the core and core-shell system determined via titration.(Sabadasch et al., 2022)

microgel system	n_{MAc} / mmol g^{-1}	apparent pK_a
core	0.71 ± 0.02	6.6 ± 0.2
core-shell	0.53 ± 0.01	6.5 ± 0.1

Table S2. Apparent reaction rate constants k_{app} derived from the linear fits of the three recycling cycles in (Fig. 6). A significant amount of catalyst was lost from cycle to cycle, indicated by the reduced residue absorbance A_i.

cycle	A_i	k_{app} / $10^{-3} s^{-1}$
1st	0.18 ± 0.01	9.2 ± 0.7
2nd	0.06 ± 0.01	4.3 ± 0.1
3rd	0.05 ± 0.02	1.6 ± 0.2

Incorporation of the UV-sensitive hydrophobic HMABP was determined by attenuated total reflection Fourier transform infrared spectroscopy (ATR-FTIR) as it provides a non-invasive method to investigate vibrational bands of aromatic comonomers. Comonomers with aromatic moieties show characteristic modes at approximately 700 cm^{-1}.(Bookhold et al., 2021) An ATR-FTIR spectrum of a dried microgel film of the core-shell system with PNIPAM-*co*-HMABP shell shows mentioned signals at 700 and 708 cm^{-1} (Fig. S1, black line).(Dirksen et al., 2022) The PNIPAM-*co*-MAc core (red line) does not show any aromatic signals in this region, indicating a successful incorporation of the UV-sensitive cross-linker into the core-shell microgels.

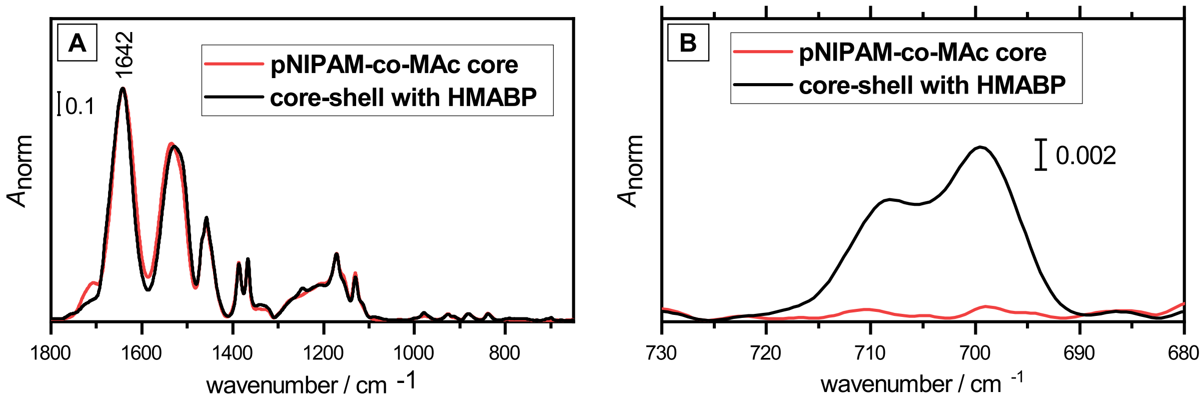

Figure S1. A: ATR-FTIR absorbance spectra of dried microgel films of the core-shell system with a PNIPAM-*co*-HMABP shell (black) and the corresponding PNIPAM-*co*-MAc core (red) between 1800 and 650 cm^{-1}. B: Enlargement of the aromatic region between 730 and 680 cm^{-1}. The absorbance was multiplied with the respective wavenumber to compensate influences by the wavenumber dependent penetration depth. The absorbance was normalized to the amide I band at 1642 cm^{-1}.

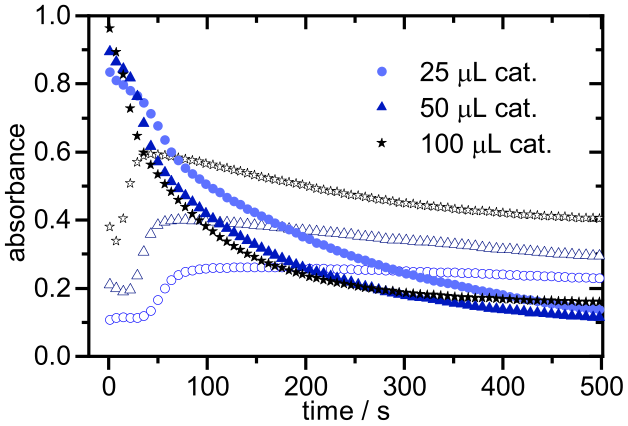

Figure S2. Absorbance at a wavelength of $300\,\mathrm{nm}$ (hollow symbols) and $400\,\mathrm{nm}$ (filled symbols) plotted against the time for catalyst amounts of 25, 50 and $100\,\mu\mathrm{L}\,\mathrm{mL}^{-1}$.

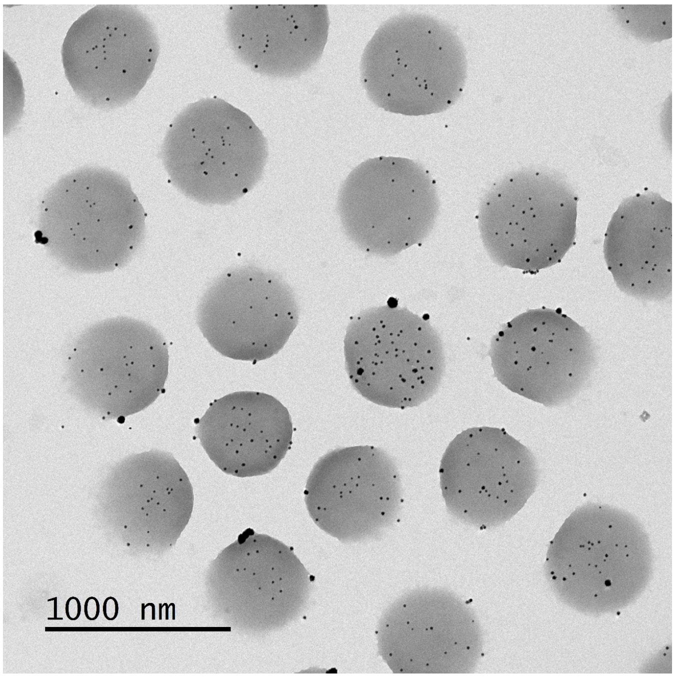

Figure S3. TEM micrographs of the loaded core-shell microgels after the first cycle and subsequent purification via centrifugation.

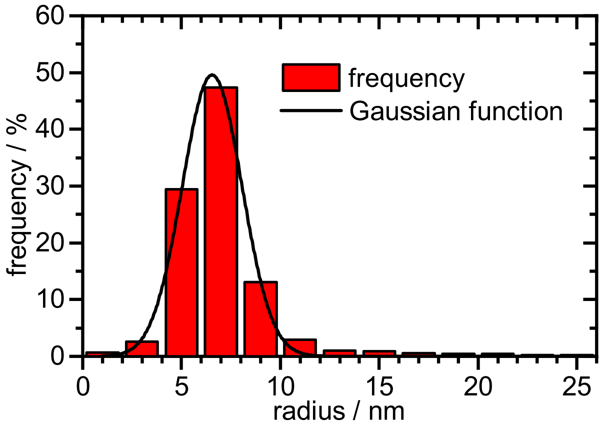

Figure S4. Size distribution of the palladium nanoparticles embedded in the core-shell system after one reaction cycle and purification process. A total of 2300 particles were analyzed and a mean particle radius of (6.5 ± 1.5) nm was obtained.

REFERENCES

Bookhold, J., Dirksen, M., Wiehemeier, L., Knust, S., Anselmetti, D., Paneff, F., et al. (2021). Smart membranes by electron beam cross-linking of copolymer microgels. *Soft Matter* 17, 2205–2214. doi:10.1039/D0SM02041A

Dirksen, M., Fandrich, P., Goett-Zink, L., Cremer, J., Anselmetti, D., and Hellweg, T. (2022). Thermo-responsive microgel-based free-standing membranes: Influence of different microgel cross-linkers on membrane function. *Langmuir* 38, 638–651. doi:10.1021/acs.langmuir.1c02195

Sabadasch, V., Dachwitz, S., Hannappel, Y., Hellweg, T., and Sewald, N. (2022). Acrylamide-based pd-nanoparticle carriers as smart catalyst for the suzuki-miyaura cross-coupling of amino acids. *Synthesis* doi:10.1055/a-1782-4224
